# Pioneer of prostate cancer: past, present and the future of *FOXA1*

**DOI:** 10.1007/s13238-020-00786-8

**Published:** 2020-09-18

**Authors:** Mona Teng, Stanley Zhou, Changmeng Cai, Mathieu Lupien, Housheng Hansen He

**Affiliations:** 1 Princess Margaret Cancer Centre, University Health Network, Toronto, Canada; 2 Department of Medical Biophysics, University of Toronto, Toronto, Canada; 3 Center for Personalized Cancer Therapy, University of Massachusetts Boston, Boston, MA 02125 USA; 4 Ontario Institute for Cancer Research, Toronto, ON Canada

**Keywords:** FOXA1, pioneer factor, transcription factor, prostate cancer, epigenetics

## Abstract

Prostate cancer is the most commonly diagnosed non-cutaneous cancers in North American men. While androgen deprivation has remained as the cornerstone of prostate cancer treatment, resistance ensues leading to lethal disease. *Forkhead box A1* (*FOXA1*) encodes a pioneer factor that induces open chromatin conformation to allow the binding of other transcription factors. Through direct interactions with the Androgen Receptor (AR), FOXA1 helps to shape AR signaling that drives the growth and survival of normal prostate and prostate cancer cells. FOXA1 also possesses an AR-independent role of regulating epithelial-to-mesenchymal transition (EMT). In prostate cancer, mutations converge onto the coding sequence and *cis*-regulatory elements (CREs) of *FOXA1*, leading to functional alterations. In addition, FOXA1 activity in prostate cancer can be modulated post-translationally through various mechanisms such as LSD1-mediated protein demethylation. In this review, we describe the latest discoveries related to the function and regulation of FOXA1 in prostate cancer, pointing to their relevance to guide future clinical interventions.

## Introduction

Prostate cancer is the most commonly diagnosed non-cutaneous cancer in North American men (American Cancer Society 2019; Canadian Cancer Society 2019), driven primarily by AR signaling for growth and survival. Given such, targeting AR signaling has been the dominant treatment strategy. Despite advances in AR pathway inhibitors, metastatic disease such as metastatic castration-resistant prostate cancer (mCRPC) and neuroendocrine prostate cancer (NEPC) arises in around 30% of patients (Grossfeld et al. 2003). mCRPC and NEPC remain lethal due to the lack of effective targeted therapies to overcome therapeutic resistance (Watson et al. 2015; Beltran et al. 2016). There is a clear need for novel strategies to manage prostate cancer, especially in the advanced metastatic setting.

**Figure 1:**
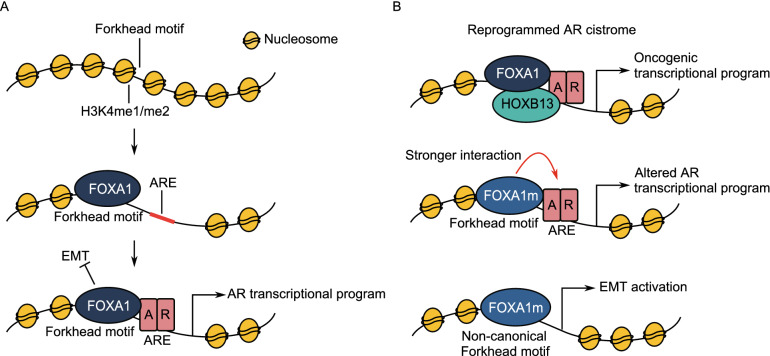
**Schematic of FOXA1 activity in (A) normal prostate and (B) prostate cancer.** (A) FOXA1 preferentially binds to genomic Forkhead motifs marked by H3K4me1/me2 histone modifications. It induces open chromatin conformation and allows for the sequential binding of AR to drive AR transcriptional programs. FOXA1 also inhibits EMT independent of AR. (B) In prostate cancer, FOXA1 can extensively reprogram AR cistrome along with HOXB13. FOXA1 mutations can result in stronger AR binding and significantly alter AR transcriptional programs. FOXA1 mutants can also induce EMT programs to drive cancer metastasis. Abbreviations: ARE, androgen-receptor response element; EMT, epithelial-mesenchymal transition

FOXA1 is a driver of prostate cancer onset and progression (Sahu et al. 2011; Gerhardt et al. 2012; Barbieri et al. 2012; Grasso et al. 2012). As a pioneer factor, it induces open chromatin conformations for the subsequent binding of lineage-specific transcription factors such as AR in prostate tissue. FOXA1 recruitment to the chromatin is facilitated by the loss of DNA methylation and the presence of histone methylation, specifically H3K4me1 and H3K4me2 modifications (Fig. 1A) (Lupien et al. 2008; Wang et al. 2009b; Sérandour et al. 2011). By directly interacting with AR to influence its recruitment to discrete genomic regions, FOXA1 regulates transcriptional programs of relevance in both normal prostate tissue and cancer (Gao et al. 2003; Sahu et al. 2013; Pomerantz et al. 2015). The silencing of *FOXA1* results in significant alteration in AR binding and gene expression (Wang et al. 2011; Sahu et al. 2011, 2013; Jin et al. 2014). In prostate tumours, FOXA1 is capable of reprogramming AR binding sites and drives oncogenic programs along with transcription factor HOXB13 (Fig. 1B) (Pomerantz et al. 2015, 2020) . In addition, FOXA1 is reported as a suppressor of neuroendocrine differentiation and its loss of expression can promote NEPC progression (Kim et al. 2017; Rotinen et al. 2018). *FOXA1* is also reported as the third most frequently mutated gene in prostate cancer (Barbieri et al. 2012). Despite these previous findings, our understanding of the mechanisms and the extent of FOXA1 involvement in prostate cancer remains incomplete. Recent studies reveal that FOXA1 is functionally altered by both coding and noncoding mutations which drives prostate cancer progression (Adams et al. 2019; Parolia et al. 2019; Gao et al. 2019; Xu et al. 2019). In addition, the regulation of FOXA1 through CREs and post-translational modifications is also revealed, opening up a new avenue for FOXA1 targeting (Zhou et al. 2020; Gao et al. 2020). In this review, we summarize the recent research on FOXA1 in prostate cancer and offer a perspective on the clinical value of FOXA1 given these findings.

### Coding mutations alter FOXA1 function

#### FOXA1 is frequently altered by coding mutations

*FOXA1* is recurrently mutated in its coding sequence in up to 9% and 13% of primary prostate cancer and mCRPC, respectively (Barbieri et al. 2012; Grasso et al. 2012; Huang et al. 2017; Adams et al. 2019; Parolia et al. 2019). In NEPC, a study of 29 tumour tissues identified single nucleotide variants (SNVs) mapped to *FOXA1* in ~25% of the tumours (Beltran et al. 2020). The frequency of *FOXA1* mutations has also been recently shown to vary with ethnic background. The analysis into the Chinese Prostate Cancer Genome and Epigenome Atlas (CPGEA) cohort revealed *FOXA1* mutation in 41% of localized prostate tumours, a rate significantly higher than Western cohorts (Li et al. 2020). Taken together, *FOXA1* is a recurrently mutated gene across various stages of prostate cancer.

**Figure 2:**
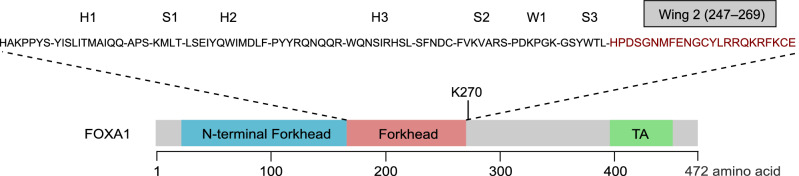
**Protein map of FOXA1 functional domains and secondary structures.** Residues in red represent Wing2 mutational hotspot. FOXA1 methylation site at lysine 270 (K270) is highlighted. Abbreviation: TA, transactivation domain

While mutations can occur throughout the *FOXA1* coding sequence, >50% of them map to the nucleotides encoding the Forkhead DNA binding domain (Clark et al. 1993; Cancer Genome Atlas Research Network 2015; Adams et al. 2019; Parolia et al. 2019; Gao et al. 2019; Xu et al. 2019; Li et al. 2020). Mutations affecting the Forkhead domain are predominantly missense point mutations and non-truncating insertions and deletions (indels) (Clark et al. 1993; Cancer Genome Atlas Research Network 2015; Adams et al. 2019; Parolia et al. 2019; Gao et al. 2019; Xu et al. 2019). In particular, the majority of mutations in the Forkhead domain cluster at nucleotides encoding residues of the second winged loop (Wing2, residues 247–269), important for DNA binding and nuclear movements (Fig. 2) (Clark et al. 1993; Sekiya et al. 2009; Cancer Genome Atlas Research Network 2015; Adams et al. 2019; Parolia et al. 2019; Li et al. 2020). Wing2 mutations are suggested to originate in the localized stage of prostate cancer and show no enrichment in metastatic diseases (Parolia et al. 2019). Beyond mutations in the Forkhead domain, truncating frameshifts account for 20% of the *FOXA1* mutations (Adams et al. 2019). They are restricted to the C-terminal half of the FOXA1 protein downstream of the Forkhead domain and are significantly enriched in metastatic prostate cancers (Adams et al. 2019; Parolia et al. 2019). The C-terminal half of FOXA1 contains an ɑ-helical region recently identified to be critical for histone interactions and chromatin opening (Iwafuchi et al. 2020). *FOXA1* coding mutations emerge throughout prostate cancer progression and cluster at hotspot regions that are important for FOXA1 protein function.

#### Coding mutations of FOXA1 lead to altered transcription factor activity

*FOXA1* coding mutations can lead to changes in DNA binding affinity and nuclear movements (Table 1). Mutations affecting the Forkhead Wing2 loop such as M253K, FENG254-7>C (deletion of residues 254 to 257, replaced by C) and F266S decreased DNA binding affinity, resulting in the global reduction of FOXA1 genomic binding (Xu et al. 2019). However, they retain the ability to form base-specific interactions at the Forkhead motifs on DNA sequence (Parolia et al. 2019; Xu et al. 2019). These FOXA1 mutants displayed a loss-of-function phenotype where they failed to rescue FOXA1-mediated gene expression upon wild-type *FOXA1* knockdown in LNCaP cells (Xu et al. 2019). In comparison, other mutants such as I176M and R261G in the Forkhead domain displayed increased nuclear movement 5–6 times compared to wild-type FOXA1 which translated to enhanced nuclear activities (Parolia et al. 2019). These mutations also led to 3–6 fold greater transcriptional activation compared to wild-type FOXA1 in luciferase reporter assays. Given the critical function of the Forkhead domain of FOXA1, mutations affecting the region can result in altered DNA binding ability and nuclear movements which lead to functional changes.

**Table 1:** Summary of key functionally validated FOXA1 coding mutations

Type	Location	Mutation	Key functional alterations	Ref
Missense	FKHD	I176M	Increased nuclear mobility; higher activation of AR signaling; increased cell growth in androgen-depleted condition	(Parolia et al. 2019)
	FKHD	R219C, R219S	Decreased transcriptional activity; increased growth of mouse organoid; preference for non-canonical FOXA1 binding motif; promotes EMT	(Adams et al. 2019)
	FKHD	D226N, D226G	Increased growth of mouse organoid; mixed results on the effect of AR activity; promotes EMT	(Adams et al. 2019; Gao et al. 2019; Xu et al. 2019)
	FKHD Wing2	H247Q, H247R, H247Y	Increased growth of mouse organoid; mixed results on the effect of AR activity; promotes EMT	(Adams et al. 2019; Gao et al. 2019)
	FKHD Wing2	M253K	Increased growth of mouse organoid; mixed results on the effect of AR activity; reduced chromatin binding; promotes EMT; increased cell growth in androgen-depleted condition	(Adams et al. 2019; Gao et al. 2019; Xu et al. 2019)
	FKHD Wing2	Y259C, Y259S	Increased transcriptional activity; increased growth of mouse organoid	(Adams et al. 2019)
	FKHD Wing2	R261G, R261C	Increased nuclear mobility; higher activation of AR signaling; increased cell growth in androgen-depleted condition; increased growth of mouse organoid	(Adams et al. 2019; Parolia et al. 2019)
	FKHD Wing2	F266L, F266S	Increased growth of mouse organoid; mixed results on the effect of AR activity; reduced chromatin binding; increased cell growth in androgen-depleted condition; promotes EMT	(Adams et al. 2019; Xu et al. 2019)
Indels	FKHD Wing2	ΔM253–N256	Increased transcriptional activity; increased growth of mouse organoid	(Adams et al. 2019)
	FKHD Wing2	ΔF254/E255	Increased transcriptional activity; increased growth of mouse organoid	(Adams et al. 2019)
	FKHD Wing2	FENG254-7>C	Reduced chromatin binding; increased cell growth in androgen-depleted condition; promotes EMT	(Xu et al. 2019)
	FKHD Wing2	ΔR265-Q271	Increased nuclear mobility; higher activation of AR signaling; increased cell growth in androgen-depleted condition	(Parolia et al. 2019)
Frameshift truncations	C-term	P358fs	Higher DNA-binding affinity; increased invasion through activation of WNT pathway	(Parolia et al. 2019)
	C-term	G275X	Increased transcriptional activity; increased growth of mouse organoid	(Adams et al. 2019)

FOXA1 mutants can remodel the accessible chromatin landscapes of prostate cancer (Table 1). Through the use of Assay for Transposase-Accessible Chromatin using Sequencing (ATAC-seq), the overexpression of wild-type *FOXA1* led to >1000 more accessible chromatin peaks in mouse prostate organoids compared to empty vector control (Adams et al. 2019). In comparison, FOXA1 Forkhead mutants such as ΔF254/E255 and R219S enhanced pioneer factor activity in comparison to wild-type, evident by the ability to induce more accessible sites in total (Adams et al. 2019). The rate of chromatin opening by the mutants was also ~5 times faster than wild-type FOXA1 (Adams et al. 2019). Although ΔF254/E255 and R219S mutants both induced large numbers of accessible sites in the genome, unsupervised clustering analysis of these sites revealed distinct patterns (Adams et al. 2019). ΔF254/E255 mutant showed accessible chromatin peaks at genomic locations similar to wild-type FOXA1. In contrast, R219S mutant induced novel accessible chromatin regions that were enriched for non-canonical Forkhead binding motif (Adams et al. 2019). Other Forkhead mutants such as D226G and M253K led to change in the preference from classic Forkhead binding motifs to non-canonical motifs (Gao et al. 2019). Along with altered ability to induce chromatin accessibility, FOXA1 mutants can induce an unique epigenetic landscape that is distinct from wild-type FOXA1 in prostate cancer.

As a pivotal regulator of AR, *FOXA1* mutations can affect FOXA1’s interaction with AR, altering androgen signaling (Table 1). However, both AR promoting and inhibiting functions of FOXA1 have been reported, revealing the complex interplay between FOXA1 and AR. Analysis of large tumour cohorts such as The Cancer Genome Atlas (TCGA) revealed that tumours with *FOXA1* mutations have a higher AR transcriptional signature compared to normal tissues and other prostate tumour subtypes (Cancer Genome Atlas Research Network 2015; Parolia et al. 2019). Mouse prostate organoid study further supported the TCGA findings as organoids expressing multiple FOXA1 mutants showed exaggerated AR-positive luminal formation (Adams et al. 2019). Conversely, other studies reported that FOXA1 mutants led to downregulation of AR signaling, suggesting an inhibitory function (Gao et al. 2019; Xu et al. 2019). In LNCaP cells expressing Forkhead mutants such as D226G, H247Y and M253K, FOXA1 and AR binding at enhancers of well-established AR-regulated genes such as *NKX3-1* and *KLK3* were significantly reduced (Gao et al. 2019). In fact, a global inhibition of AR binding at both FOXA1-dependent and -independent sites was reported (Xu et al. 2019). These mutants may prevent AR binding to chromatin owing to their increased interaction with AR but decreased chromatin binding ability (Fig. 1B) (Gao et al. 2019; Xu et al. 2019). As a result, *NKX3-1*, *KLK3* and other AR-related genes were found significantly downregulated in cells expressing FOXA1 mutants (Gao et al. 2019). FOXA1 mutations resulted in different AR signaling patterns. Both inhibitory and promoting functions were reported and the activity may vary based on the mutations and models used in the study.

Studies on the coding mutations of *FOXA1* have revealed distinct activities in terms of regulating AR signaling and open chromatin landscapes (Adams et al. 2019; Parolia et al. 2019; Gao et al. 2019; Xu et al. 2019; Hankey et al. 2020). FOXA1 mutants may contribute to specific stages of prostate cancer progression given their divergent activities due to mutations.

#### Coding mutations in FOXA1 promote EMT and metastasis

While FOXA1 promotes cell proliferation through AR, it also possesses an AR-independent function of inhibiting EMT and cell invasion (Fig. 1A) (Jin et al. 2013). 22RV1 prostate cancer cells expressing FOXA1 C-terminal truncation mutants showed an accumulation of transcriptionally active β-catenin, indicative of WNT pathway activation (Parolia et al. 2019). Consequently, these cells displayed 2–3 fold increased invasion *in vitro* and greater ability to metastasize *in vivo* in zebrafish embryos (Fig. 1B) (Parolia et al. 2019). Mechanistically, EMT activation by C-terminal truncation mutants may be due to the loss of interaction with TLE3 which is a WNT repressor (Parolia et al. 2019). Several missense and non-truncating indel Forkhead mutants also demonstrated similar ability to induce EMT and metastasis (Jin et al. 2013; Adams et al. 2019; Gao et al. 2019; Xu et al. 2019). In particular, RNA sequencing of mouse prostate organoids expressing FOXA1 mutant R219S revealed an enrichment of EMT genes which was not observed with other Forkhead mutants such as D226N and ΔF254/E255 (Adams et al. 2019). R219S is enriched in NEPC compared to prostate adenocarcinoma, a subtype in which EMT is implicated (Adams et al. 2019). EMT was also activated in CWR22-RV1 mCRPC cells expressing D226G, H247Y and M253K FOXA1 Forkhead mutants (Gao et al. 2019). It remains unclear, however, how these FOXA1 mutants induce EMT, whether it is a loss of ability to inhibit EMT or a gain of function of the mutants to directly induce gene expression changes (Xu et al. 2019). Despite some missing knowledge, EMT inhibition may be a novel strategy to treat metastatic prostate cancer with *FOXA1* mutations. The ability of FOXA1 mutants to activate EMT may play a role in prostate cancer metastasis and progression in certain types such as NEPC.

### Noncoding mutations converge on FOXA1

#### Mutations converge onto FOXA1 cis-regulatory landscape to drive oncogenic expression

The genome is organized through three-dimensional chromatin looping interactions between CREs (Dixon et al. 2012, 2016; Nora et al. 2012; Szabo et al. 2019). The careful organization of these interactions leads to cell-type specific gene expression programs. In cancer, mutations including SNVs and structural variants (SVs) can alter the activity and interactions between CREs to drive aberrant gene expression, which is seen in the case of *FOXA1*.

**Figure 3:**
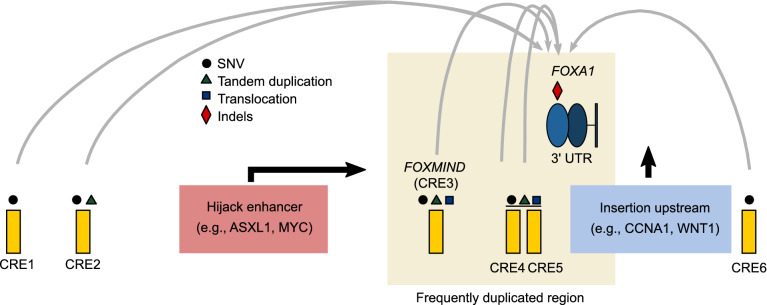
**Noncoding mutations converge onto FOXA1.** *FOXA1* locus is frequently altered by structural rearrangements inclusive of translocations and tandem duplications. Translocations can result in genes hijacking the activity of the *FOXMIND* enhancer. In addition, translocations can insert oncogenes upstream of *FOXA1*. Along with *FOXMIND*, 5 additional functional CREs harbouring SNVs are identified near FOXA1 locus which can affect the expression of *FOXA1*. The 3′ UTR region of *FOXA1* is frequently mutated with indels being the dominant type of mutation. Abbreviations: SNV, single nucleotide variant; 3′ UTR, 3′ untranslated region; CRE, *cis*-regulatory elements

*FOXA1* expression is altered by noncoding mutations across prostate cancer stages. Functional studies revealed 6 CREs involved in regulating *FOXA1* mRNA expression (Fig. 3) (Parolia et al. 2019; Zhou et al. 2020) with each being targeted by mutations. Consistent with the essentiality of *FOXA1*, the deletion of each CRE of the plexus resulted in significant reduction in *FOXA1* mRNA expression and LNCaP prostate cancer cell growth (Zhou et al. 2020). In primary prostate cancer, 6 SNVs from 5/200 patients (2.5%) were mapped to the 6 CREs. These SNVs were shown to have gain-of-function activity through modulating the binding of AR, FOXA1, HOXB13 and SOX9 *in vitro* (Zhou et al. 2020). Consistent with the importance of *FOXA1 cis*-regulatory landscape in disease progression, SVs were found to target loci harbouring the CREs in up to 30% of mCRPC tumours (Parolia et al. 2019). Together with coding mutations, it was estimated that the cumulative frequency of FOXA1 alterations is over 34% in mCRPC (Parolia et al. 2019). The SVs affecting *FOXA1* predominantly consist of translocation and tandem duplication and are significantly enriched in mCRPC (Parolia et al. 2019). Two types of translocation events involving the *FOXA1* CREs emerge in these tumours. The first involves the repositioning of genes from other chromosomes (i.e., *ASXL1*, *ERG*, *ETV1*, *MYC*, and *SKIL*) into the proximity of a *FOXA1* CRE termed *FOXMIND* (Fig. 3) (Quigley et al. 2018; Parolia et al. 2019). The second type of translocation involves the insertion of potent oncogenes, namely *WNT1*, *HOXA1* and *CCNA1*, upstream of the *FOXA1* promoter. Both types of translocation events hijacked the *cis*-regulatory activity of *FOXA1* CREs to significantly upregulate the oncogenic expression of these genes, including *FOXA1* in mCRPC tumours (Parolia et al. 2019). Tandem duplications within the *FOXA1* locus account for 70% of all SVs identified across >1,400 primary and advanced prostate tumours (Parolia et al. 2019). They predominantly flank the loci harbouring *FOXMIND-FOXA1* (Fig. 3) as *FOXA1* and *FOXMIND* are co-duplicated in 89% of the tumours with tandem duplications, driving aberrant *FOXA1* and *MIPOL1* mRNA expression (Parolia et al. 2019). These efforts highlight that the *cis*-regulatory plexus of FOXA1 can be recurrently hijacked in prostate tumours to drive aberrant gene expression and disease progression.

#### Noncoding mutations alter the binding of FOXA1 genome-wide

Germline single-nucleotide polymorphisms (SNPs) and somatic SNVs can modulate FOXA1 genomic binding and transcription regulation. Genome-Wide Association Studies (GWAS) identified more than 160 risk loci to be associated with prostate cancer susceptibility (Farashi et al. 2019) with the majority of SNPs map to the noncoding genome (Maurano et al. 2012). These SNPs have the ability to confer disease risk through modulating the binding of transcription factors such as FOXA1 to CREs (Zhang et al. 2014; Zhou et al. 2016). For instance, the rs1859961 variant allele being a G at the 17q24.3 risk locus can significantly decrease FOXA1 binding intensity at the active CRE driving *SOX9* oncogenic expression (Zhang et al. 2012). Similar findings were observed at the 8q24 prostate cancer risk locus where rs183373024 can abolish FOXA1 binding (Hazelett et al. 2013). Expanding on this concept, prostate cancer risk SNPs have the propensity to pose allele-specific binding towards AR-FOXA1 complexes (Whitington et al. 2016). Genome-wide analysis of prostate cancer risk SNPs and somatic SNVs demonstrated that they converge and enrich at the binding sites of other master transcription factors besides FOXA1, including AR, HOXB13 and SOX9 (Ahmed et al. 2017; Mazrooei et al. 2019). Deeper analyses revealed that the >270,000 somatic SNVs identified from 200 primary prostate tumours (Fraser et al. 2017; Espiritu et al. 2018) were enriched at tumour-specific and shared binding sites of these master transcription factors, but not at benign-specific binding sites (Mazrooei et al. 2019). While most of these SNVs are likely passengers (Sabarinathan et al. 2016; Mazrooei et al. 2019), a small subset of these SNVs can significantly increase FOXA1 binding at CREs that regulate *MYC* (Mazrooei et al. 2019) and *FOXA1* (Zhou et al. 2020) itself. These SNVs and SNPs add another layer of complexity whereby FOXA1 confers prostate cancer complexity to the regulation of FOXA1 cistrome.

### Post-translational modifications alter FOXA1 activity

In addition to transcriptional regulations, FOXA1 activity can also be modulated post-translationally through acetylation, phosphorylation, methylation, and the addition of small ubiquitin-like modifiers (SUMO) (Kohler and Cirillo 2010; Sutinen et al. 2014; Yamaguchi et al. 2017; Gao et al. 2020). FOXA1 is reported to be acetylated by histone acetyltransferase p300 at 11 putative lysine residues, of which 5 of them are clustered within the Forkhead domain. Acetylation can attenuate FOXA1 binding to DNA and reduce its ability to remodel chromatin (Kohler and Cirillo 2010). Similarly, the addition of SUMO to lysine residues of FOXA1 can also result in downregulation of its transcriptional activity and nuclear mobility (Sutinen et al. 2014). Furthermore, phosphorylation by c-Abl is also reported, highlighting that FOXA1 is subjected to various post-translational modifications (Yamaguchi et al. 2017). Recently, methylation mediated by Lysine specific demethylase 1 (LSD1) has been identified as an additional mechanism that regulates FOXA1 activity and holds clinical potential as an avenue to target FOXA1 (Gao et al. 2020). LSD1 is known as a transcriptional repressor that demethylates enhancer-associated H3K4me1/me2 (Shi et al. 2004). In addition to its repressor function, LSD1 is implicated in prostate cancer as a coactivator of AR signaling (Metzger et al. 2005; Wissmann et al. 2007; Cai et al. 2014). Upon *LSD1* knockdown with siRNA in LNCaP cells, androgen-stimulated genes were broadly suppressed (Cai et al. 2014). ChIP-seq analysis revealed that the LSD1 genomic binding sites largely associate with FOXA1 and active enhancer marks (Cai et al. 2014). Through immunoprecipitation, FOXA1 was found to physically interact with LSD1 in an AR-independent manner (Cai et al. 2014). Although LSD1 is best characterized as a histone demethylase, it can also demethylate nonhistone proteins (Huang et al. 2007; Wang et al. 2009a). A recent study showed that LSD1 is capable of demethylate K270 residue of FOXA1 (Gao et al. 2020). This residue is located at the carboxyl-end of the Wing2 region and has been shown to be important for the FOXA1 interaction with core histones (Fig. 2) (Iwafuchi et al. 2020). The demethylation of FOXA1 K270 by LSD1 is required for its optimal binding to the chromatin. Upon LSD1 inhibition, a larger fraction of FOXA1 remained methylated at K270 which led to decreased global binding and ability to induce open chromatins. As a result, AR signaling was significantly altered due to impaired FOXA1 pioneer activity (Cai et al. 2014; Gao et al. 2020). The regulation of FOXA1 activity remained unclear for many years which contributed to the difficulty of targeting FOXA1 as a therapeutic target. The recent knowledge that FOXA1 activity can be regulated by post-translational modifiers such as LSD1 opens up a novel avenue to target FOXA1.

### From discovery to clinical applications

While recent reports provided new exciting insights on FOXA1, questions regarding their clinical utility remain. Recent studies looking at the implications of *FOXA1* across different stages of prostate cancer revealed new functional roles relevant for prognosis, therapeutic response and targeted therapy.

#### Chemically antagonizing FOXA1 and downstream pathways

Transcription factors such as FOXA1 are known to be difficult to inhibit directly due to their nuclear localization and mechanisms of action (Dang et al. 2017). Modern technologies are expanding our toolkits to inhibit FOXA1 from other angles. As case and point, a drug screen comprising 550 drugs across three breast cancer cell lines identifies CDK1 as a therapeutic target for FOXA1 inhibition (Wang et al. 2018). In the same vein, numerous FDA-approved drug libraries are commercially available for researchers to evaluate new inhibitors for previously undruggable targets. Academic consortia such as Structural Genomic Consortium (Arrowsmith et al. 2012; Scheer et al. 2019; Wu et al. 2019) also offer chemical probes that directly inhibit epigenetic factors implicated in FOXA1 regulation (Lupien et al. 2008; Sérandour et al. 2011; Müller et al. 2018). An example is the new finding that LSD1 regulates FOXA1 activity which implies the use of LSD1 inhibitors may be a viable approach to antagonize FOXA1 activity in prostate cancer (Gao et al. 2020). The LSD1 inhibitors such as GSK2879552 and SP-2509 have shown effectiveness in suppressing prostate tumour cell growth with and without the use of enzalutamide in combination in preclinical animal models (Sehrawat et al. 2018; Gao et al. 2020). In addition, targeting LSD1 presents an advantage as several inhibitors such as IMG-7289 and ORY-1001 are currently in phase 2 clinical trials for essential thrombocythemia, acute myeloid leukemia and other diseases (Fang et al. 2019). Adding LSD1 inhibitors in combination with existing therapies such as enzalutamide may achieve synergistic effect in prostate cancer control (Gao et al. 2020). In addition to epigenetic factors, PARP-2 involved in DNA damage repair pathways may also be a therapeutic target as it is reported as an interactor of FOXA1 that facilitates AR recruitment and transcription (Gui et al. 2019).

Targeting FOXA1-regulated pathways such as EMT may also be a treatment strategy in combination with antiandrogen therapies. *FOXA1* downregulation is shown to lead to TGF-β signaling and EMT activation which can be blocked by TGF-β receptor I inhibitor LY2157299 (Song et al. 2019). The use of LY2157299 demonstrated synergistic effects in combination with enzalutamide in effectively suppressing tumour growth and metastasis in mCRPC preclinical mouse models (Song et al. 2019). Similarly, FOXA1 Forkhead coding mutations such as D226G, H247Y and M253K can also result in EMT activation. Consequently, cells expressing these mutant FOXA1 were sensitive towards MET inhibitor crizotinib treatment in mCRPC xenograft models, which targets the c-Met protein known to induce EMT (Gao et al. 2019). Altogether, targeting FOXA1-regulated pathways such as EMT can be another avenue to improve disease control especially for the metastatic cases where EMT is implicated.

Considering the prevalence and differential FOXA1 activity conferred by specific *FOXA1* mutations, future studies may consider conducting inhibition-screens with FOXA1 mutant model systems. The recent development of prostate mouse organoids (Adams et al. 2019) that harbor FOXA1 mutants shows promise as a powerful resource towards identifying mutant-specific FOXA1 inhibitors. This is especially important to maximize the therapeutic index of drugging FOXA1 by minimizing toxicity as FOXA1 is also expressed in normal tissues. This ultimately potentiates the idea of precision oncology, selecting patients for tailored therapy based on their *FOXA1* mutation status.

#### Tracking prostate cancer using FOXA1 related mutations

*FOXA1* mutations found across various stages of prostate cancer present an opportunity to track disease progression. Their inclusion in targeted sequencing panels from bodily fluids (urine or blood) can serve as a means to track minimal residual disease since normal tissues should not exhibit FOXA1 mutations. A targeted sequencing of 72 prostate cancer driver genes in the plasma cell-free DNA of patients with mCRPC revealed 37 somatic mutations mapped to *FOXA1* 3′ UTR in 34/290 (12%) of the patients (Fig. 3) (Annala et al. 2018). In fact, *FOXA1* harboured the highest 3′ UTR mutation rate among all 72 genes of interest with indels being the dominant type of alteration (Annala et al. 2018). These indels can be found in the early stage of prostate tumours and are not present in other cancer types, such as bladder cancer, indicating prostate cancer specificity (Annala et al. 2018). The study demonstrated that *FOXA1* mutations can be readily detected in liquid biopsy and can potentially serve as a prostate cancer-specific biomarker. Recent findings have shown the differential prevalence of *FOXA1* mutations across prostate cancer stages, for instance the early emergence of Forkhead Wing2 coding mutations in localized disease and the enrichment of truncation mutations and SVs in metastatic diseases (Adams et al. 2019; Parolia et al. 2019). This further supports the notion that mutation status surrounding *FOXA1* can inform disease progression. Based on the varying function of FOXA1 mutants, the prognostic value of different FOXA1 mutations should be further evaluated in future studies. Through identifying inhibitors that antagonize specific FOXA1 mutants, this approach potentiates personalized treatment. With the constant cellular tumour cellular turnover releasing cell-free DNA, liquid biopsy for *FOXA1* mutations permits longitudinal tracking for residual disease and early detection for recurrence.

## Summary

Recent functional characterization of FOXA1 mutants shed light on their complex modes of action in driving prostate cancer where the function may be divergent depending on the residues affected. Overlaying the prostate cancer epigenome reveals a catalog of CREs critical for *FOXA1* mRNA expression that are extensively perturbed through various modes of somatic mutations. In addition, post-translational modifications such as protein demethylation mediated by LSD1 are identified as additional regulations of FOXA1 activity. Deeper understanding of the somatic mutations and regulatory mechanisms may yield novel biomarkers and therapeutic strategies complementary to our existing standard of care with the ultimate goal of improving patient outcomes. With present day high-throughput methodologies and model systems that have been lacking in the past, there is promise in translating the past and recent biological findings regarding FOXA1 into clinical utility.
